# Apathy in Lewy body disease and its effects on functional impairment over time

**DOI:** 10.3389/fneur.2024.1339190

**Published:** 2024-01-18

**Authors:** Carolyn W. Zhu, Hillel T. Grossman, Gregory A. Elder, Howie Rosen, Mary Sano

**Affiliations:** ^1^Brookdale Department of Geriatrics and Palliative Medicine, Icahn School of Medicine at Mount Sinai, New York, NY, United States; ^2^James J. Peters VA Medical Center, Bronx, NY, United States; ^3^Department of Psychiatry, Alzheimer Disease Research Center, Icahn School of Medicine at Mount Sinai, New York, NY, United States; ^4^Department of Neurology, Icahn School of Medicine at Mount Sinai, New York, NY, United States; ^5^Department of Neurology, Memory and Aging Center, University of California San Francisco (UCSF), San Francisco, CA, United States

**Keywords:** Lewy body disease, apathy, function, parkinsonism, longitudinal study

## Abstract

**Background and objectives:**

Apathy strongly affects function in Alzheimer’s disease and frontotemporal dementia, however its effect on function in Lewy Body Disease (LBD) has not been well-described. This study aims to (1) examine the prevalence and persistence of apathy in a large, national cohort of well-characterized patients with LBD, and (2) estimate the effect of apathy on function over time.

**Methods:**

Study included 676 participants with mild cognitive impairment (MCI) or dementia in the National Alzheimer’s Coordinating Center Uniform Data Set. Participants were followed for an average of 3.4 ± 1.7 years and consistently had a primary diagnosis of LBD. Apathy was defined by clinician judgment, categorized into four mutually exclusive profiles: (1) never apathetic across all visits, (2) at least one but <50% of visits with apathy (*intermittent apathy*), (3) ≥50% but not all visits with apathy (*persistent apathy*), and (4) *always apathy* across all visits. Dementia severity was measured by baseline Clinical Dementia Rating score. Parkinsonism was defined by the presence of bradykinesia, resting tremor, rigidity, gait, and postural instability. Functional impairment was assessed using the Functional Assessment Questionnaire (FAQ).

**Results:**

Baseline characteristics of the sample were: average age = 72.9 ± 6.9, years of education = 15.6 ± 3.4, Mini Mental State Exam (MMSE) = 24.4 ± 5.4, Geriatric Depression Scale (GDS) = 3.8 ± 3.2, FAQ = 12.0 ± 9.1. 78.8% were male and 89% were non-Hispanic white. Prevalence of apathy increased from 54.4% at baseline to 65.5% in year 4. 77% of participants had apathy at some point during follow-up. Independent of cognitive status and parkinsonian features, FAQ was significantly higher in participants with intermittent/persistent and always apathetic than never apathetic. Annual rate of decline in FAQ was faster in participants who were always apathetic than never apathy.

**Discussion:**

In this large national longitudinal cohort of LBD patients with cognitive impairment, apathy was strongly associated with greater functional impairment at baseline and faster rate of decline over time. The magnitude of these effects were clinically important and were observed beyond the effects on function from participants’ cognitive status and parkinsonism, highlighting the importance of specifically assessing for apathy in LBD.

## Introduction

Apathy is increasingly recognized as one of the most prevalent and disabling behavioral symptoms in neurocognitive disorders and can have profound consequences for morbidity, mortality, quality of life, caregiver burden, healthcare costs and progression of cognitive impairment (1–8). Recently published diagnostic criteria for apathy in neurocognitive disorders address conditions from mild cognitive impairment (MCI) to dementia across etiologies (9). Dementia with Lewy bodies (DLB) is a neurocognitive disorder in which Lewy bodies containing alpha-synuclein protein are deposited in neurons of the cerebral cortex (10). DLB is the second most common form of degenerative dementia after Alzheimer’s disease (AD), accounting for up to 20% of all dementia cases in the US (11), and is associated with substantial burden including higher and earlier mortality (12, 13), higher healthcare costs (14), and lower quality of life (15). As in other neurocognitive disorders, apathy is one of the most frequent and stable behavioral symptoms in DLB (16–18). Current estimates of prevalence of apathy in DLB range from 35 to 100% (16, 19–22). Variability in reported rates is likely due to differences in samples and instruments used to assess apathy.

Our understanding of the relationship between apathy and functional decline in patients with DLB across the spectrum of cognitive impairment is limited. Existing studies often include small number of patients with DLB, use diagnostic and inclusion criteria that are inconsistent, and emphasize late-stage outcomes (17, 18, 23, 24). To fill in some of the gaps, we use the National Alzheimer’s Coordinating Center Uniform Data Set (NACC-UDS) to (1) examine the prevalence and persistence of apathy in participants with clinically-diagnosed DLB and (2) estimate the relationship between apathy and functional decline over time across the spectrum of cognitive impairment.

The NACC-UDS is a national database of all research participants who enrolled in one of the 39 past and present National Institute on Aging (NIA)-funded Alzheimer’s Disease Research Centers (ADCs) located throughout the US. While the database was not specifically designed to study DLB, the ADCs routinely enroll patients with many non-AD related dementias including vascular dementia, frontotemporal dementia (FTD), and DLB. It has the unique advantage of being one of the largest cohorts of research participants who have had comprehensive and standardized evaluations that include cognition, function, behavioral, and affective symptomatology as well as a robust diagnostic process from neurologic and neuropsychiatric experts in major tertiary medical centers across the US (25). The cohort of participants in the NACC-UDS have a wide range of cognitive status and have been followed up for an average of 4 years. Additionally, similar methodology has been used to examine apathy in AD and FTD (26, 27), facilitating comparisons of apathy across dementia subtypes at the same centers using the same standardized tools.

DLB is grouped with Parkinson’s disease (PD) under the umbrella of Lewy body disease (LBD), and is defined by the timeframe over which parkinsonian features appear in relation to cognitive changes. If cognitive changes precede or occur within 1 year of the appearance of parkinsonism, the condition is considered DLB. In contrast, if cognitive changes occur in the setting of well-established PD (typically greater than 1 year) then the condition is considered Parkinson’s disease with dementia. Guidelines for clinical diagnoses are specified in the NACC-UDS Guidebook^1^ (28, 29). Because the NACC-UDS has a rich set of characterizing variables, we aimed to explore whether the effects of apathy on function were consistent in the presence of parkinsonian motor features.

## Materials and methods

### Data source and sample derivation

Data used in the current study were drawn from NACC-UDS between September 2005 (start date of the UDS) and the December 2022 data freeze (*N* = 47,164) (30). Recruitment, participant evaluation, and diagnostic criteria have been detailed elsewhere (31). Briefly, beginning in September 2005, participants have been followed prospectively from 42 past and present NIA-funded Alzheimer’s Disease Centers (ADC) located throughout the US. All ADCs enroll and follow participants at approximately 12-month intervals with standardized protocols and provide data for research to NACC-UDS. Written informed consent were provided by all participants and their informants and approved by local Institutional Review Boards (IRBs). Research using the NACC-UDS was approved by the University of Washington IRB. This study followed the STROBE reporting guideline.

**Figure 1:**
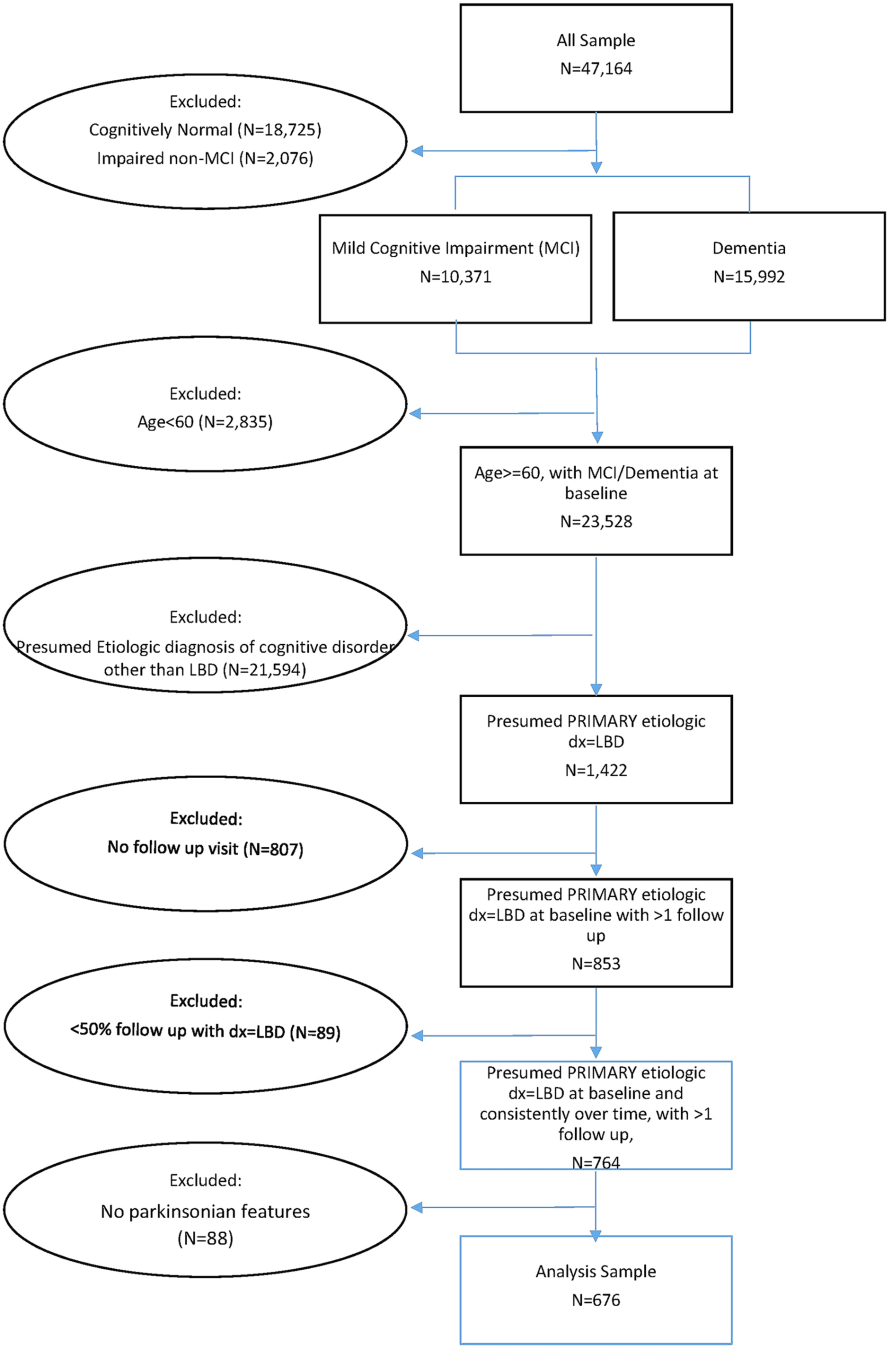
Sample selection flow chart.

At each visit, participants’ cognitive status was assessed using a standardized neuropsychiatric battery and categorized as cognitively normal, impaired not-MCI, MCI, or dementia based on up-to-date research diagnostic criteria, utilizing all available information according to UDS procedures, including patient exam, informant-provided history, cognitive testing, neuroimaging, and genetic profile (32). Diagnosis of LBD follows the McKeith criteria described in the NACC-UDS Guidebook (28). Clinician-observed parkinsonian features were assessed for all participants regardless of cognitive status. We selected our analysis sample as follows: First, we included individuals with MCI (*N* = 10,371) or dementia (*N* = 15,992) (9). Among these participants, we identified those aged 60 or older with a primary diagnosis of LBD at baseline who had at least one follow-up visit (*N* = 853). We then examined the consistency of the participant’s LBD primary diagnosis over time and identified 89 participants whose primary diagnosis was not LBD in more than half of their follow-up visits. We excluded these participants with inconsistent LBD diagnosis over time. Finally, we excluded a small number of participants who did not have any parkinsonian signs and who was MCI at baseline but reverted to normal cognition or impaired non-MCI. Sample Selection Flow Chart is in Figure 1.

### Measures

#### Apathy

At each visit, clinicians assessed whether the participant “manifests meaningful change in behavior-apathy, withdrawal” following the NACC-UDS protocol based on all available information including clinical assessment, informant report derived from the Neuropsychiatric Inventory (NPI) (33, 34) and medical records review. Based on this clinician judgment of apathy, we constructed indicators for the presence of apathy at a visit and examined cumulative prevalence (proportion of participants with an endorsement of apathy in at least one visit over the entire follow-up period). Persistence of apathy between two consecutive visits *v* and *v + 1* was defined by the proportion of participants who had an endorsement of apathy at visit *v* and also had an endorsement of apathy at visit *v + 1*. Based on how often a participant was reported to have apathy throughout the follow-up period, we categorized participants into four mutually-exclusive profiles: (1) those who never had apathy across all visits (*never apathy*), (2) those with intermittent apathy (at least one but <50% of visits with apathy, *intermittent apathy*), (3) those with persistent apathy (≥50% visits with apathy, *persistent apathy*), and (4) those who always had apathy in all visits (*always apathy*).

#### Function

Our main dependent variable was participants’ function, measured using the Functional Assessment Questionnaire (FAQ) reported from interviews with study partners, a validated measure of function targeted for older adults with normal cognition, MCI, and dementia appropriate for clinical and home settings as well as for research (35, 36). Total FAQ score was divided by the number of tasks attempted to obtain a standardized score (37).

#### Dementia severity

Dementia severity was measured based on baseline CDR Sum of Boxes (CDR-SB) score: mild (CDR-SB score 0–0.5); moderate (CDR-SB score 1–4); severe (CDR-SB score ≥ 4.5), according to validated threshold values for identifying dementia in patients (38).

#### Demographic and clinical characteristics

Demographic characteristics included age, sex, race/ethnicity, years of education, marital status, and living alone (yes/no). Participant medical history was obtained by clinician interview and review of medical records as reported to NACC-UDS. Depressive symptoms were measured using the 15-item Geriatric Depression Scale (GDS-15) (39, 40). Medication use was reported using a medication inventory (Form A4) which included all medications (including nonprescription drugs, vitamins, and supplements) taken by the participant within 2 weeks of their visit. We examined utilization of medication for AD symptoms, antiparkinsonian, antidepressant, antipsychotic, and anxiolytic, sedative, or hypnotic agent.

#### Parkinsonism features

Clinician-observed parkinsonian features were assessed for all participants regardless of cognitive status. Measures of parkinsonian features varied across NACC-UDS versions. Based on parkinsonian features that were consistently evaluated in the NACC-UDS (presence of bradykinesia, rigidity, resting tremor, parkinsonian gait disorder, and postural instability, Form B3 in UDSv1-2, Form B8 in UDSv3), we constructed a summary score of the number of parkinsonian features (range = 0–5) as a proxy for severity of parkinsonism.

### Statistical analyses

Multivariable estimation of the effects of apathy on function was performed using linear mixed models (LMM). Our main independent variables were *apathy group* (reference group: *never apathy*), time, and their interactions. Coefficients on *apathy group* estimated differences in FAQ scores at baseline for each *apathy group* compared to *never apathy*. We hypothesized that worse *apathy groups* would be associated with worse FAQ. The coefficient for time, measured using UDS visit, estimated overall change in FAQ over time. We included a squared term for time to estimate the overall rate of change over time. The interaction terms between *apathy groups* and time estimated differences in the rate of change in FAQ over time between *apathy groups* compared to *never apathy*. A positive coefficient indicated faster decline in FAQ over time in each *apathy group* compared to *never apathy*.

To estimate the independent effect of *apathy group* on function, the following variables were included in the estimation models: (1) baseline cognitive status (MCI vs. dementia) and its interaction terms with time, (2) number of parkinsonian features present and its interaction terms with time, (3) indicators for taking medications for AD symptoms, antiparkinsonian agents, antidepressant, antipsychotic, and anxiolytic, sedative, or hypnotic agent as well as total number of medications used; and (4) demographic and clinical characteristics including baseline age, sex, being non-Hispanic white, education, GDS, years of follow-up, and indicator for UDS version. All models also included participant level random intercept and slope to allow differences at baseline and over time between participants. A random effect for ADC (i.e., which center evaluated the participant) was included to allow nesting of participant within each ADC. All analyses were performed using Stata 16.0 (41). Statistical significance was set *a priori* at *p* < 0.05.

We performed several sensitivity analyses to examine the stability of the results between apathy profile and function: (1) using participant’s baseline CDR-SB instead of cognitive status, (2) using a NACC reported diagnosis of Parkinson’s disease instead of parkinsonian features, (3) using a subsample of participants with at least 2 follow-up visits. In all of these sensitivity analyses, results on the relationship between apathy and function were substantively the same as our main estimation model. Results of these sensitivity analyses are available upon request.

## Results

### Baseline sample characteristics

At baseline, average age of the cohort was 72.8 ± 6.9, 80.2% male, 89.8% non-Hispanic white, with 15.6 ± 3.3 years of schooling (Table 1). 67% of the participants had dementia, 33% MCI. Average MMSE was 24.1 ± 5.5 and GDS was 4.0 ± 3.2. Participants reported taking 7.2 ± 4.1 medications. Anti-dementia (60.6%), antiparkinsonian (45.9%), antidepressant (45.9%), anxiolytic, sedative, hypnotic (24.5%), and antipsychotic (14.4%) medications were commonly reported. Mean number of parkinsonian signs was 3.5 ± 1.2. Majority of participants reported bradykinesia (88.9%), gait (81.4%), rigidity (76.0%), and postural instability (72.7%). Total FAQ score was 12.8 ± 9.1. More than half of the participants reported having difficulties in each FAQ item, ranging from 49% reporting difficulties with using the stove, to 86% reporting difficulties with assembling tax records, business affairs, or papers. Participants were followed for an average of 3.4 ± 1.7 years.

**Table 1:** Baseline characteristics by clinician judgment of apathy.

Variables	All Sample		No apathy		Apathy		Value of *p*
*N*	676		308		368		
Age, mean (SD)	72.8	(6.9)	72.9	(6.8)	72.8	(6.9)	0.675
Male (%)	80.2		77.6		82.3		0.124
Non-Hispanic white (%)	89.8		91.2		88.6		0.258
Years of education, mean (SD)	15.6	(3.3)	15.7	(3.3)	15.6	(3.3)	0.563
**Cognitive status (%)**							
MCI	33.4		46.4		22.6		<0.001
Dementia	66.6		53.6		77.4		
CDR Sum of Boxes, mean (SD)	4.6	(3.7)	3.6	(3.4)	5.5	(3.8)	<0.001
≤0.5 (%)	5.9		10.4		2.2		<0.001
1–4 (%)	49.4		59.7		40.8		
≥4.5 (%)	44.7		29.9		57.1		
MMSE, mean (SD)	24.1	(5.5)	25.0	(5.0)	23.4	(5.9)	<0.001
GDS, mean (SD)	4.0	(3.2)	3.5	(2.8)	4.5	(3.4)	<0.001
Number of medications taken, mean (SD)	7.2	(4.1)	7.3	(4.1)	7.1	(4.2)	0.651
Anti-dementia medications (%)	60.6		55.7		64.7		0.018
Any antiparkinsonian medications (%)	45.9		50.8		41.7		0.018
Antidepressant (%)	45.9		40.0		50.8		0.005
Anxiolytic, sedative, hypnotic agent (%)	24.5		22.3		26.4		0.221
Antipsychotic (%)	14.4		10.5		17.8		0.008
**Parkinsonian signs (%)**							
Bradykinesia	88.9		85.5		91.8		0.010
Gait	81.4		78.1		84.1		0.049
Rigidity	76.0		72.7		78.8		0.065
Postural instability	72.7		68.8		75.9		0.042
Resting tremor	33.9		33.8		34.0		0.956
Number of parkinsonian signs, mean (SD)	3.5	(1.2)	3.3	(1.3)	3.6	(1.2)	0.010
Years of follow up, mean (SD)	3.4	(1.7)	3.4	(1.5)	3.4	(1.8)	0.101
FAQ, mean (SD)	12.8	(9.1)	10.1	(8.6)	15.0	(8.9)	<0.001

Apathy was present in 54.4% of the participants at baseline. Compared to those without apathy, those with apathy were more likely to have dementia, with higher GDS, lower MMSE, greater functional impairment, and had more parkinsonian signs (all *p* < 0.01). Although total number of medications reported were similar, participants with apathy were more likely to report taking anti-dementia medications, anti-depressants, and anti-psychotic medications, but less likely to be taking antiparkinsonian medications (all *p* < 0.02). There were no statistically significant differences between participants with and without apathy in demographic characteristics or follow up time.

### Prevalence and persistence of clinician judgment of apathy over time

The proportion of participants with a clinician endorsement of apathy at a visit ranged between 54% at baseline and 66% at visit 4 (Table 2). Cumulative prevalence of apathy (i.e., proportion of participants with an endorsement of apathy in at least one visit over the entire follow-up period) was 77%. Persistence of apathy between two consecutive visits (i.e., proportion of participants who had an endorsement of apathy at two consecutive visits) increased over time from 45% between visit 1 and 2, 48% between visit 2 and 3, to 54% between visits 3 and 4. Throughout the entire follow-up period, 23% of participants never had an endorsement of apathy from the clinicians, 11% had intermittent apathy, 28% had persistent apathy, and 39% always had apathy throughout all visits.

**Table 2:** Prevalence and persistence of clinician-judged apathy.

	All sample
**A. Prevalence (%)**	
Presence at a visit	
v1	54.4
v2	56.8
v3	64.8
v4	65.5
Cumulative prevalence	76.9
**B. Persistence (%)**	
Visit to visit persistence	
v1–v2	45.0
v2–v3	47.5
v3–v4	54.0
**C. Apathy group (%)**	
Never apathy across all visits	22.6
Intermittent apathy (<50% of all visits)	11.1
Persistent apathy (≥50% of all visits)	27.5
Always apathy	38.8

### Estimated relationships between apathy and function

**Figure 2:**
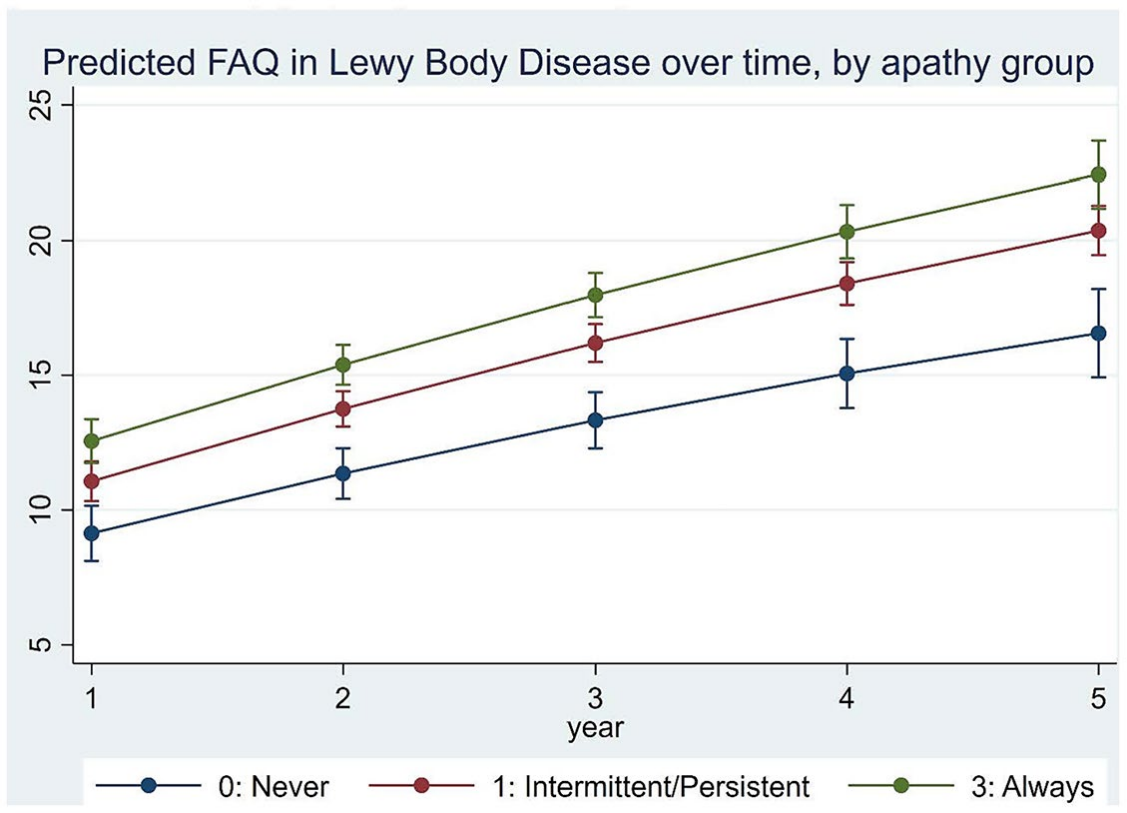
Predicted FAQ by apathy group over time.

Adjusted LMM estimation results on the relationships between apathy and function over time are in Table 3. Figure 2 plots model-predicted FAQ scores for different apathy groups over time. Results show that FAQ scores worsened by an average of 3.336 ± 0.455 points per year with a slowing rate of decline by−0.120 ± 0.041 points each year (both *p* < 0.001).

**Table 3:** Mixed effects regression estimates of the relationships between apathy group, cognitive status, parkinsonism and functional decline over time.

Variables	Coefficient estimate	SE	*p*	95% CI	
**Overall rate of worsening over time**					
Year	3.336	(0.455)	<0.001	2.445	4.227
Year*Year	-0.120	(0.041)	0.003	−0.201	−0.040
**Baseline differences by apathy group, cognitive status, and parkinsonism**					
Apathy group (Reference: never apathy)					
Intermittent/persistent apathy	1.459	(0.911)	0.109	−0.327	3.246
Always apathy	2.810	(0.936)	0.003	0.975	4.645
Cognitive status (Reference: MCI)	9.145	(0.724)	<0.001	7.725	10.565
Number of parkinsonian signs	0.558	(0.279)	0.045	0.012	1.104
**Longitudinal effects of apathy group, cognitive status, and parkinsonism (interactions with time)**					
Apathy group (Reference: never apathy)					
Intermittent/persistent apathy	0.470	(0.295)	0.111	−0.108	1.047
Always apathy	0.612	(0.236)	0.040	0.027	1.251
Cognitive status (Reference: MCI)	−0.257	(0.203)	0.207	−0.655	0.142
Number of parkinsonian signs	−0.174	(0.086)	0.062	−0.342	0.006
**Other covariates**					
Baseline age	0.126	(0.038)	0.001	0.050	0.201
Male	−2.863	(0.664)	<0.001	−4.164	−1.562
Non-Hispanic white	−1.102	(0.864)	0.202	−2.794	0.591
Years of education	−0.010	(0.080)	0.902	−0.168	0.148
Years of follow up	−0.574	(0.171)	0.001	−0.909	−0.239
GDS	0.183	(0.056)	0.001	0.072	0.293
Number of medications	0.138	(0.095)	0.147	−0.049	0.324
Taking any antiparkinsonian medications	0.151	(0.393)	0.702	−0.619	0.921
Taking anti-dementia medications	0.583	(0.395)	0.140	−0.191	1.356
Taking antipsychotic	1.368	(0.481)	0.004	0.426	2.310
Taking antidepressant	0.210	(0.369)	0.569	−0.513	0.933
Taking anxiolytic, sedative, hypnotic agent	0.646	(0.390)	0.098	−0.119	1.411

Models also included NACC-UDS version, participant level random intercept and slope to allow participants differences at baseline and overall rate of change over time. A random effect for ADC was also included to allow nesting of subjects within each ADC.

Compared to never apathy, FAQ scores were 2.810 ± 0.936 points higher at baseline in those who were always apathetic (*p* < 0.001). Over time, rate of decline was 0.612 ± 0.236 point per year faster in those always apathetic (*p* < 0.05) compared to never apathy. Baseline differences and rate of decline in FAQ were not statistically significantly different between those with intermittent/persistent apathy and never apathy.

Compared to those with MCI, FAQ scores in participants with dementia was 9.145 ± 0.724 points higher at baseline (*p* < 0.001). Rate of decline over time was not statistically significantly different between dementia and MCI. Each additional parkinsonian signs was associated with 0.558 ± 0.279 points higher FAQ scores at baseline (*p* < 0.05). Other variables that were associated with higher FAQ scores included older age, being male, worse GDS scores, and taking antipsychotic medications.

## Discussion

In this study, we examined apathy profile and its association with functional impairment in a large cohort of extensively characterized patients with a diagnosis of LBD across the spectrum of cognitive impairment followed for an average of 4 years. Results showed that apathy was common in LBD even in the mild stages of cognitive impairment, with higher prevalence in patients with more severe cognitive impairment. Our results adds to prior studies of apathy in DLB that have reported wide-ranging estimates of the prevalence of apathy in DLB from 35% to 100% (16, 19–22). Comparisons of findings across studies are difficult because most studies have enrolled small numbers of patients, ranging from 20 to 200 (16, 17, 19–22) and had short follow-up periods of a year or two (24).

Rates of apathy observed in this LBD cohort are best considered in comparison to earlier works derived from the same dataset in patients with AD and bvFTD. In these three cohorts from the NACC-UDS, rates of apathy in LBD (74%) were higher than previously published results in AD (60%) but lower than in bvFTD (88%) (27, 42). Persistence of apathy in LBD also was higher than in AD but lower than in bvFTD during similar length of follow-up time. More than a third of the participants with LBD had apathy at all visits, compared to 17% in AD and 50% in bvFTD. There are differences in the characteristics between these cohorts but they are as would be expected. For example, the LBD cohort was younger than the AD cohort but older than the bvFTD cohort. Clinically, the LBD cohort had better cognition at baseline than both AD and bvFTD cohorts. At the same time, the LBD cohort had worse function than AD but better than bvFTD.

Independent of a range of participant demographic and clinical characteristics, we observed an average rate of decline in function by 3.336 ± 0.455 points per year, which is consistent with others who reported annual rate of decline in LBD of 1–4 points per year (23, 25). Our results added to the literature by showing more persistent apathy strongly associated with greater functional impairment at baseline and with a faster rate of functional decline. Specifically, compared to the never apathy group, FAQ at baseline was 2.810 ± 0.936 points higher in the always apathy group, and 1.459 ± 0.911 points higher in the intermittent/persistent apathy group. In addition, compared to the never apathy group, FAQ increased by 0.612 ± 0.236 points faster each year in the always apathy group, suggesting accelerated decline in function in this group. Translating these estimated differences into predicted FAQ for each group, results suggest differences in FAQ between always apathy and never apathy groups increased from 3.4 points at baseline to 5.9 points at visit 5. Currently there are no guidelines on whether these differences would be considered clinically important for LBD. However, in therapeutic trials for AD, a 3–5 point increase in FAQ in a year are considered clinically meaningful decline (43). It is worth noting that these estimated independent effects of apathy on function were similar to early reports in bvFTD and AD (26, 42). Specifically, in AD patients, FAQ declined by almost one point per year faster in those with intermittent apathy and 1.3 points faster in persistent apathy as well as in those who were always apathetic (26). In bvFTD patients, compared to participants without or with intermittent apathy, FAQ declined by half a point per year faster in those with persistent and almost one point per year faster in those with always apathy (42). It also is worth noting that all three sets of analyses controlled for depression in the estimation models, so that the effects of apathy with increased functional decline over time was distinct from depression. The similarity of these estimates across several neurocognitive disorders provide support to the new diagnostic criteria for apathy in neurocognitive disorders addressing conditions from MCI to dementia across etiologies (9).

In the current study, patient’s function was measured using the FAQ, which includes items of instrumental activities of daily living ranging from preparing meals to managing personal finances. These tasks are often provided by patient’s family, friends, and other informal caregivers. While not directly examined in this paper, others have reported apathy associated with substantial caregiver stress and burden (44, 45). Our results suggest that efforts in reducing apathy not only may be beneficial to patient’s function but also may have additional benefit to caregivers.

Pharmacological and non-pharmacological interventions for apathy in LBD are complex and under-investigated (4, 46). Evidence of efficacy of non-pharmacological interventions are not robust (47). While no drugs are currently approved to treat apathy, several pharmacological treatment options for apathy in patients with LBD have been suggested (48). For example, rivastigmine has been shown to reduce apathy in a group of clinically characterized patients with DLB (49). Our study highlight the importance of apathy as an outcome in LBD in our continued efforts in finding treatment options.

In the current LBD cohort, the effects of apathy on function were observed independent of effects from cognitive status and parkinsonism. As would be expected, baseline functional status was worse for participants with dementia than MCI, but the rate of functional decline over time was not statistically significantly different between dementia and MCI. We also observed that the number of parkinsonian features were associated with worse function at baseline, but was not associated with faster rate of functional decline. Because parkinsonian features can be found in other dementing conditions including frontotemporal dementia and vascular dementia and also can be the result of neuroleptic exposure, which is common in patients with dementia, we consider the changes captured in NACC-UDS data reflective of parkinsonism but not necessarily Parkinson’s disease. Nevertheless, we explored whether using a NACC-UDS recorded diagnosis of Parkinson’s disease instead of parkinsonian features affected our estimation results. Estimated relationship between apathy and function were unchanged, increasing our confidence in our estimation results.

Our study has several limitations. First, we defined apathy using clinician judgment. While NACC does not dictate or format how ADCs conduct clinical assessments, NACC requires the ADCs conduct full clinical assessments in order to gather information and report clinical judgments to NACC-UDS via standardized data forms. The expectation is that clinician judgment is made by one with expertise in neurodegenerative and neuropsychiatric diseases, based on all available clinical information from research visits, including detailed history taking, medical record review, neurologic and neuropsychiatric exam, and information collected from the Neuropsychiatric Inventory Questionnaire (NPI-Q) as part of the NACC-UDS assessment. While the NPI-Q is a well-validated and accepted instrument that includes an assessment of apathy, we consider clinician judgment a more comprehensive measure as it incorporates a much broader range of data than the informant-reported NPI-Q as the NPI-Q is limited to the informant’s interpretation of participant’s behaviors. We believe that in the absence of criteria-based diagnosis, clinician judgment of apathy from ADC experts may represent best practices across the US. We explored correlation between clinician judgment of apathy and the NPI-Q in the current sample. Results showed that among those who had a clinician judgment of apathy, 87% also were reported as having apathy on the NPI-Q. These results are similar to earlier reports that showed high correlation between clinician judgment of apathy and the NPI-Q in AD (7), which strengthens confidence in these results. The dataset does not include sufficient information to apply the newly developed diagnostic criteria for apathy in neurocognitive disorders. Future work that uses newer consensus diagnostic criteria to replicate this study will be needed.

Second, while we controlled in the current analysis a set of medications that may influence functional impairment or apathy, it should be noted that medications reported in the NACC-UDS are limited to self-reported medications use for the 2-week window preceding each annual visit. Medication use between visits is unknown and may have changed throughout the year, and reasons for treatment are not reported. Third, the participants in this study are a clinic-based research cohort that is overwhelmingly non-Hispanic white and highly educated, which may not reflect patients with LBD in real-world clinics, limiting our ability to examine potential differences across racial/ethnic, socioeconomic, and education groups. Lastly, although we focused on apathy, which is one of the most common behavioral symptoms of dementia, we did not examine the comprehensive set of behavioral symptoms captured in NACC-UDS.

Strengths of this report include the large sample size and long follow-up of the cohort, inclusion of broad range of severity in cognitive impairment, and extensive clinical characterization of participants including neuropsychological testing and functional assessment. Although this research sample may not be representative of more typical patient populations, diagnoses were made by ADC-based dementia specialists, neurologists, and/or geriatricians using standardized structured instruments and criteria. Diagnosis of DLB specifically follows the 2017 McKeith criteria^2^ (28, 29). These characterizations of patients likely represent current best practices in the field. Additionally, common data fields within the NACC-UDS allow comparison of results across dementia etiologies, broadening the implication of our results.

In summary, apathy is recognized as a prevalent and disabling behavioral feature in many neurocognitive disorders and can have profound effects on personal, social and occupational functioning in patients and their caregivers. Our report shows a high prevalence and persistence of apathy in LBD, greater impairment, and a faster rate of decline in functional status in the presence of apathy. These effects are clinically important and are consistent and independent of the effect on functional status of cognitive impairment and dementia and severity of parkinsonism. Findings highlight the importance of targeting the treatment and management of apathy in LBD.

## Data availability statement

Publicly available datasets were analyzed in this study. The data that support the findings of this study are openly available at: https://naccdata.org/data-collection/forms-documentation/uds-3.

## Ethics statement

Research using the NACC-UDS was approved by the University of Washington IRB. The studies were conducted in accordance with the local legislation and institutional requirements. Written informed consent for participation was not required from the participants or the participants’ legal guardians/next of kin in accordance with the national legislation and institutional requirements.

## Author contributions

CZ: Conceptualization, Data curation, Formal analysis, Investigation, Methodology, Visualization, Writing – original draft, Writing – review & editing. HG: Conceptualization, Funding acquisition, Investigation, Methodology, Writing – review & editing. GE: Conceptualization, Funding acquisition, Investigation, Methodology, Writing – review & editing. HR: Investigation, Methodology, Writing – review & editing. MS: Conceptualization, Funding acquisition, Investigation, Methodology, Writing – review & editing.

## Footnotes

^1^ https://naccdata.org/data-collection/training

^2^ https://naccdata.org/data-collection/training
